# Detección de coproantígenos de *Echinococcus granulosus* en canes de trabajadores de camales y comercializadores de vísceras en Lima metropolitana

**DOI:** 10.26633/RPSP.2017.10

**Published:** 2017-02-08

**Authors:** Veronika Merino, Néstor Falcón, Noelia Morel, Gualberto González

**Affiliations:** 1 Facultad de Medicina Veterinaria y Zootecnia Universidad Peruana Cayetano Heredia Lima Perú Facultad de Medicina Veterinaria y Zootecnia, Universidad Peruana Cayetano Heredia, Lima, Perú.; 2 Comisión Nacional de Zoonosis Comisión Nacional de Zoonosis Ministerio de Salud Pública Montevideo Uruguay Comisión Nacional de Zoonosis, Ministerio de Salud Pública, Montevideo, Uruguay.; 3 Cátedra de Inmunología de la Facultad de Química Instituto de Higiene, Universidad de la República Montevideo Uruguay Cátedra de Inmunología de la Facultad de Química, Instituto de Higiene, Universidad de la República, Montevideo, Uruguay.

**Keywords:** *Echinococcus granulosus*, zoonosis, equinococosis, perros, Perú, *Echinococcus granulosus*, zoonoses, echinococcosis, dogs, Peru

## Abstract

**Objetivo.:**

*Demostrar la presencia de* Echinoccocus granulosus *en el hospedero definitivo en la ciudad de Lima, Perú, mediante la detección de antígenos del parásito en heces de canes pertenecientes a trabajadores y comercializadores de vísceras de centros de beneficio autorizados en Lima metropolitana*.

**Métodos.:**

*Se recolectaron muestras de heces de 58 canes, que fueron evaluadas utilizando la técnica coproELISA para detectar antígenos secretorio/excretorio de* E. granulosus*. Mediante una encuesta se obtuvo información sobre las prácticas de alimentación y el manejo de las mascotas*.

**Resultados.:**

*El 13,8% (8/58) de canes fue positivo a* E. granulosus*. En 27,8% (5/18) de los hogares se encontró al menos un animal positivo y se estimó que en las familias que tenían más de cuatro canes las posibilidades de encontrar al menos uno positivo eran mayores. En todos los hogares con al menos un can positivo sus mascotas se alimentaban con vísceras. El 94,4% (17) de los participantes no tenía conocimiento de las formas de contagio de la equinococosis*.

**Conclusiones.:**

*Los resultados muestran la presencia de hospederos definitivos en la zona urbana de Lima y subrayan la necesidad de aumentar la difusión de las prácticas para evitar la transmisión del parasito*.

## Abstract (en)

**Objective.:**

To demonstrate the presence of *Echinoccocus granulosus* in the definitive host in the city of Lima, Perú, by detecting parasite antigens in the stool of dogs belonging to offal handlers and merchants in authorized slaughterhouses in Metropolitan Lima.

**Methods.:**

Stool samples were collected from 58 dogs and examined using the copro-ELISA technique for the detection of secretory/excretory antigens of *E. granulosus*. A survey was conducted to obtain information on pet feeding and handling practices.

**Results.:**

Positivity to *E. granulosus* was detected in 13.8% (8/58) of the dogs. In 27.8% (5/18) of the homes, at least one animal showed positivity, and in families that had more than four dogs the chances of finding positivity in at least one dog were higher (P < 0.05). In all homes where at least one dog tested positive the pets were fed on offal. Of study participants, 94.4% (17) knew nothing about the routes of transmission of hydatid disease.

**Conclusions.:**

Results show the presence of definitive hosts in the urban area of Lima and underscore the need to more widely disseminate practices for the prevention of parasite transmission.

La equinococosis quística (EQ) es una infección producida por *Echinococcus granulosus* (1). Los canes (hospederos definitivos) albergan la forma adulta del parásito y el ganado (hospedero intermediario), la forma larvaria denominada quiste hidatídico. El hombre está expuesto a infectarse con huevos de *E. granulosus* al estar en contacto con un perro infectado o encontrarse en un ambiente contaminado (2), convirtiéndolo en hospedero aberrante (1, 3). Una vez en el ser humano, el parásito es transportado por vía linfática y sanguínea hacia distintos órganos donde formará quistes y causará la enfermedad llamada equinococosis quística humana (EQH) (3). Esta infección es un problema de salud pública a nivel mundial y reviste especial importancia en regiones productoras de ovinos (4, 5), como gran parte de los países de América del Sur (6).

La incidencia de EQH es mayor en zonas rurales endémicas de la sierra central de Perú (7). Sin embargo, en los últimos años, se ha notificado en zonas urbanas no endémicas la presencia de casos de EQH (8, 9), así como de equinococosis canina (8, 10). Una de las técnicas más efectivas de diagnóstico y tamizaje de *E. granulosus* en perros es la detección de coproantígenos mediante ELISA, ya que puede detectar la infección durante el período prepatente con sensibilidad y especificidad elevadas (92,6 y 86,4%, respectivamente) (3, 11–13).

La presencia de casos de EQH autóctona en zonas urbanas de Lima sugiere la presencia de un ciclo de transmisión *E. granulosus* en la ciudad. Las mascotas de los trabajadores de centros de beneficio pueden representar una población particularmente expuesta debido al desconocimiento de las causas y consecuencias de esta enfermedad (14, 15). Por tal razón, el objetivo de este estudio fue evaluar la presencia de *E. granulosus* en canes pertenecientes a matarifes y comercializadores de vísceras de tres centros de beneficio en Lima Metropolitana utilizando la prueba de coproELISA para su diagnóstico. Los centros de beneficio incluidos en el estudio son centros autorizados por el Servicio Nacional de Sanidad Agraria (SENASA) y, de acuerdo con lo establecido por el Reglamento Sanitario del Faenado de Animales de Abasto (16), cuentan con un médico veterinario oficial o autorizado por el SENASA, que realiza labores de inspección y evaluación de dichos centros y sus procesos.

## MATERIALES Y MÉTODOS

Los centros de beneficios que formaron parte del estudio fueron contactados a través de sus representantes administrativos, quienes autorizaron el ingreso del grupo de estudio a los centros y la interacción con sus trabajadores.

Se realizó un estudio observacional y transversal para estimar prevalencia de *E. granulosus* en muestras de heces de caninos pertenecientes a trabajadores de mataderos o comercializadores de vísceras en la ciudad de Lima. El tamaño de muestra para realizar el estudio se calculó mediante la fórmula de detección de la enfermedad utilizando las siguientes restricciones: nivel de confianza del 95% y prevalencia límite que se quería detectar de 5%. El tamaño de la muestra calculado fue de 58 canes y el muestreo se realizó por conveniencia y agotamiento. Los animales incluidos en el estudio pertenecen a 18 matarifes y comercializadores de vísceras de 3 centros de beneficio autorizados en la ciudad de Lima Metropolitana, donde se realiza el faenamiento de animales de abasto, que incluyen bovinos caprinos, ovinos y porcinos procedentes de la sierra central del país. Los centros de beneficio se identificaron como A, B y C.

La recolección de muestras de heces fue realizada por cada dueño de los canes incluidos en el estudio. A cada participante se le entregó un kit de recolección de heces caninas previamente preparado, que incluía un envase de plástico de boca ancha, un par de guantes desechables, una cuchara de plástico desechable y bolsas de plástico. Cada participante recibió una breve explicación sobre el contenido del kit y una demostración sobre cómo recolectar heces de sus perros de forma adecuada. El dueño fue instruido, una vez con los guantes puestos, a recoger con ayuda de la cuchara de plástico aproximadamente 10 gramos de heces y colocarlos en el recipiente que contenía una suspensión buffer para la preservación de la muestra (25 mL PBS – 1% formalina – 0,3% Tween 20).

Los dueños que decidieron entregar muestras, recibieron, a guisa de incentivo por su participación, una dosis de antiparasitario (Praziquantel 5 mg/kg y Pyrantel 15 mg/kg) para cada una de sus mascotas. Por otro lado, se recolectó información sobre conocimientos y prácticas potencialmente asociadas con infecciones por *E. granulosus* en canes y de EQH en humanos. Estas encuestas se llevaron a cabo antes de las sesiones informativas (sobre EQH) para personas que mostraron interés en participar en el estudio.

Las muestras fecales recolectadas se prepararon siguiendo el método descrito por Morel, et al. (13). El sobrenadante obtenido del procesamiento de cada una de las muestras se separó y envió a la Cátedra de Inmunología de la Facultad de Química del Instituto de Higiene de la Universidad de la República (UDELAR) de Uruguay, para procesarlas con la técnica ELISA sandwich para detectar coproantígenos específicos. Se empleó el kit diagnóstico descrito previamente (13), que desarrollaron la Comisión Nacional de Zoonosis de Uruguay y la Cátedra de Inmunología de la Facultad de Química de la UDELAR.

La presencia de al menos una muestra positiva indica que la infección por *E. granulosus* en esta población se encontraría en la prevalencia límite fijada para el estudio. Se consideró hogar positivo a todo aquel que tuviera al menos una mascota positiva a *E. granulosus*. Al encontrarse muestras positivas, se calculó su frecuencia y se evaluó su asociación con las variables tipo de actividad del dueño (trabajador de camal o comerciante de vísceras), lugar de procedencia (distrito), y costumbres de alimentación de los perros (alimentar con vísceras, cocción de las vísceras). Los porcentajes se compararon con la prueba de Chi cuadrado.

El protocolo de investigación fue verificado y aprobado por el Comité Institucional de Ética de la Universidad Peruana Cayetano Heredia. Todos los dueños de los perros participantes en el estudio dieron su consentimiento informado verbal. A todos ellos se les informó de la posibilidad de retirarse del estudio en cualquier momento. La identificación de los participantes y los resultados obtenidos se mantuvieron en el anonimato y no se utilizaron identificadores de los materiales de estudio.

## RESULTADOS

En el estudio participaron 18 (62,1%) trabajadores de un total de 29 que fueron sensibilizados al inicio del estudio. Del total de participantes, 11 (61,1%) eran matarifes y 7 (39,9%), comercializadores de vísceras. De las 58 muestras colectadas, 8 (13,8%) fueron positivas a coproantígenos de *E. granulosus*. El número de propietarios que tenían cuando menos un animal positivo fue 5 (27,8%). La distribución de las muestras positivas según el centro de beneficio y el tipo de actividad de sus propietarios aparece en el cuadro 1. No se encontró ninguna asociación entre dichas variables y la presencia de casos positivos.

**CUADRO 1.** Distribución de muestras de heces de canes positivos a Echinococcus granulosus según el lugar de trabajo y el tipo de actividad del propietario. Lima, 2012

Variable del estudio	Estrato de la variable	Muestras recolectadas (No.)	Muestras positivas	
			No.	%
Centro de beneficio	A	28	3	10,7
	B	17	3	17,7
	C	13	2	15,4
Tipo de actividad	Matarife	47	5	10,6
	Comercio de vísceras	11	3	27,3

En el cuadro 2 se muestra la distribución de los casos positivos considerando el hogar del propietario como unidad de observación. Los casos de hogares positivos se distribuyeron según el centro de beneficio donde trabajaba el propietario del can, el tipo de actividad que realizaba, el tiempo desarrollando la actividad, y el número de animales que poseía. Sólo se encontró una asociación entre el número de animales en el hogar y la presencia de hogares positivos (*P* < 0,05) y las familias que tenían más de cuatro canes en casa fueron las que tenían mayor posibilidad de encontrar al menos un animal infectado con *E. granulosus*.

**CUADRO 2.** Distribución de hogares con canes positivos a *Echinococcus granulosus* según el lugar de trabajo, el tipo y el tiempo de actividad y la tenencia de canes del propietario. Lima, 2012

Variable de estudio	Estrato de la variable	Total de hogares (No.)	Hogares positivos	
			No.	%
Centro de beneficio	A	7	3	42,9
	B	7	1	14,3
	C	4	1	25,0
Tipo de actividad	Matarife	11	4	36,4
	Comercio de vísceras	7	1	14,3
Tiempo dedicado a la actividad	< 5 años	2	0	0
	5-10 años	5	4	80,0
	> 10 años	5	1	20,0
	No contestó	6	0	0
Tenencia de canes	1 can	7	0	0
	2-4 canes	6	1	16,7
	> 4 canes	5	4	80,0

En total, 15 (83,3%) los participantes admitieron alimentar a sus mascotas con carne y vísceras, aunque no indicaron de qué especie, y sólo 5 (27,8%) reconocieron que los alimentaban con carne o vísceras crudas. Todos los hogares en los cuales se encontró al menos un animal positivo, reconocieron que los alimentaban con vísceras. Por otro lado, 17 (94,4%) participantes no tenían conocimiento de las formas de contagio de la equinococosis.

## DISCUSIÓN

El presente estudio pone de manifiesto la presencia de canes positivos a *E. granulosus* entre las mascotas de trabajadores de centros de beneficio, lo cual confirma la presencia del hospedero definitivo de EQH en la zona urbana de Lima y en una población específica.

La presencia de *E. granulosus* en zona urbana ya se había notificado con anterioridad. En un estudio se demostró la presencia de canes infectados en otras zonas urbanas de Chincha, ciudad localizada al sur de la capital (8). En otros dos estudios se estimó que 5,1% de los canes en la periferia de un camal en Lima (17) y 18% de los canes de 10 camales no autorizados en la misma ciudad serían hospederos definitivos del parásito (10). En otra investigación se estimó que 7,9 % de casos de EQH en niños fueron pacientes que, según las pruebas epidemiológicas reunidas, nunca salieron de la zona urbana (9).

El alcance de este estudio no permite definir con certeza cuál es la fuente de infección de las mascotas de los trabajadores. Sin embargo, dicha fuente podría estar asociada con la actividad de sus propietarios. Es probable que los animales tengan acceso a vísceras infectadas obtenidas del centro de trabajo de los dueños. Por ello, se baraja la hipótesis según la cual la probabilidad de que las mascotas de los trabajadores relacionados con centros de beneficio estuvieran infectadas por *E. granulosus* sería mayor.

Si bien el Reglamento Sanitario del Faenado de Animales de Abasto establece en su Anexo N°13 que la hidatidosis es una enfermedad de notificación obligatoria, y que además es causa de condena parcial (vísceras afectadas), esto no siempre es acatado por los trabajadores de los centros de beneficio (16). En su actividad es común observar que durante el beneficio el quiste hidatídico se desecha en contenedores simples de basura, lo que constituye un incumplimiento de la indicación de separar vísceras con quistes y de incinerarlas posteriormente, como estipula el Artículo N° 71 del Reglamento Sanitario del Faenado de Animales de Abasto (16). Esta práctica podría estar favoreciendo el uso de las vísceras desechadas para la alimentación de los canes, lo cual ya había sido considerado por otros investigadores que sugireron que el manejo deficiente de vísceras durante y después del beneficio favorecería el acceso de los canes a los quistes hidatídicos (14). No obstante, la disponibilidad de estos quistes no estaría limitada a los canes que ingresan o habitan en los camales, sino que los trabajadores de estos centros de beneficio estarían trasladando estas vísceras a sus hogares para alimentar a sus mascotas.

Es posible que estos trabajadores opten por alimentar a sus mascotas con carne y vísceras de desecho, porque ello representa una menor inversión económica en comparación con el uso de alimentos equilibrados. Esto se haría más evidente cuando el número de mascotas que han de mantenerse es mayor, puesto que al requerir una mayor cantidad de alimento la inversión económica aumenta y ello obliga a los propietarios a buscar alternativas para disminuir gastos. El elevado número de canes que conviven en un hogar representaría un factor de riesgo importante de la equinococosis canina.

En este estudio se observó que 80% de las familias que fueron positivas tenían 4 o más canes, un hallazgo que concuerda con lo demostrado por otros investigadores en un estudio de casos y controles realizado con pacientes diagnosticados de EQH en el Departamento de Cirugía del Hospital Nacional Hipólito Unanue en Lima (18). Sin embargo, las personas no suelen reconocer que utilizan estos desechos del camal para alimentar a sus mascotas, porque conocen la prohibición de extraer vísceras del centro de beneficio. Esto pude explicar que en este estudio sólo 27,7% de los entrevistados admitió alimentar con carne o vísceras crudas a sus canes.

El peligro de exposición a *E. granulosus* para las personas aumenta al permitir que sus mascotas defequen en sus casas (en el patio, el jardín o el baño) o en sus alrededores (parques, veredas y jardines exteriores). Como todos los casos positivos en este estudio son canes a los cuales se permite este tipo de comportamiento, las familias estarían constantemente expuestas a los huevos de *E. granulosus*.

Otra práctica negativa que perpetúa la infección por *E. granulosus* es la falta de desparasitación periódica de las mascotas. Ninguno de los trabajadores entrevistados había administrado antiparasitarios a sus mascotas en los últimos seis meses. La desparasitación periódica con fármacos antiparasitarios de amplio espectro es una práctica preventiva que interrumpe el ciclo de transmisión de esta zoonosis.

El desconocimiento del mecanismo de transmisión de la equinococosis en los animales y en el hombre fue evidente entre los participantes del estudio. Sólo 1 de los 18 participantes conocía que el can estaba involucrado en el ciclo de transmisión. El desconocimiento de estos temas es un factor más que influye en las prácticas de alimentación y en el manejo de las mascotas y favorece la transmisión y la diseminación de la hidatidosis. Por ello, si se tiene como objetivo la prevención y el control de esta enfermedad, se debe de iniciar un programa de educación sanitaria dirigido especialmente a las poblaciones más vulnerables y en riesgo.

Por otro lado, para diseñar programas de control efectivos, es necesario identificar de manera eficiente la presencia del parásito en hospederos definitivos. El diagnóstico *de E. granulosus* ha sido un inconveniente en Perú, porque no se ha implantado una prueba de tamizaje rápida y sencilla.

En este estudio se empleó la técnica de detección de coproantígeno ELISA desarrollada por la Cátedra de Inmunología de Facultad de Química de la Universidad de la República de Uruguay, que está siendo validada para su uso en estudios epidemiológicos por el Ministerio de Salud de Perú (13). Esta prueba se considera la mejor opción para el diagnóstico de *E. granulosus* en poblaciones de canes, debido a que puede detectar coproantígenos, incluso durante el período de prepatencia, como se señaló anteriormente (11, 13). Asimismo, reduce los riesgos biológicos a que podrían estar expuestos el personal y el ambiente, pues no los expone directamente a huevos infecciosos como lo hacen las técnicas de necropsia y purga con arecolina. Además, es una prueba relativamente económica en comparación de otras como la PCR o el Western Blot, lo cual permite su uso masivo en grandes poblaciones de estudio (3, 12).

Considerando los resultados de este estudio y la importancia de esta zoonosis, es necesario actuar en el ciclo de transmisión de la equinococosis quística. Si bien es cierto que el ciclo no se reproduce a nivel urbano con la misma intensidad que en áreas rurales, sus consecuencias sobre la salud de las personas pueden ser mayores por el estrecho contacto con las mascotas que tienen en ellas, especialmente en los niños que viven en zonas urbanas (5). Por ello, es necesario que las autoridades adopten las medidas de control correspondientes para evitar que esta enfermedad siga propagándose. Es necesario también que se promueva y asegure el cumplimiento de las leyes viegentes, tales como la que regula el Régimen Jurídico de Canes, que establece que “*por razones de salud pública está prohibido el ingreso, permanencia o tenencia de canes a establecimientos como camales o mataderos…”* y “*Todo propietario y criador de canes, está obligado a: no alimentarlos con desechos o productos contaminados o en descomposición* […] *y no permitir que hurguen en la basura*” (19).

Estas medidas de control y prevención deben interrumpir el ciclo de transmisión en distintos puntos evitando que los canes ingieran vísceras contaminadas; desparasitando a los canes en riesgo; controlando el beneficio de ganado y asegurando la eliminación adecuada de los desechos; brindando educación sanitaria a la población expuesta a EQH, e incentivando el cambio de actitud y prácticas, sobre todo en los niños que, por sus hábitos, costumbres y relación con las mascotas, representan una población particularmente vulnerable.

Dos de las principales limitaciones del estudio son la poca confianza que la población de estudio tenía en el equipo de investigación y el potencial sesgo de deseabilidad social. Estos factores afectaron el volumen de las muestras disponibles y de la información que se obtuvo, así como a la poca disposición a responder a las preguntas, incluso en el grupo de personas interesadas en participar en él. Sin embargo, permite demostrar la presencia de canes infectados con *E. granulosus* pertenecientes a matarifes y comercializadores de vísceras, lo que subraya la importancia de realizar estudios epidemiológicos de esta enfermedad para conocer más su dinámica e impacto en poblaciones específicas en riesgo.

Por ultimo, el estudio pone de manifiesto la presencia de canes positivos a coproantígenos de *E. granulosus* entre las mascotas caninas en la ciudad de Lima. Tener más de cuatro canes por familia y alimentar a estas mascotas con vísceras serían variables que favorecerían la infección por equinococosis canina.

### Agradecimiento.

Este estudio no hubiera sido posible sin la invaluable colaboración y donación de los kits de diagnóstico por parte de la Cátedra de Inmunología de la Facultad de Química de la Universidad de la República en Uruguay. Los autores expresan un agradecimiento especial a M. V. Henry W. Hernández Isla, de la Estrategia Sanitaria de Zoonosis del Instituto Nacional de Salud del Niño de Perú, por su importante contribución al estudio.

### Financiación.

El estudio se financió con fondos propios.

### Declaración.

Las opiniones expresadas por los autores son de su exclusiva responsabilidad y no reflejan necesariamente los criterios ni la política de la Organización Panamericana de la Salud o de la *RPSP/PAJPH*.
